# Synergistic effect of surface phosphorylation and micro-roughness on enhanced osseointegration ability of poly(ether ether ketone) in the rabbit tibia

**DOI:** 10.1038/s41598-018-35313-7

**Published:** 2018-11-15

**Authors:** Naoyuki Fukuda, Masayuki Kanazawa, Kanji Tsuru, Akira Tsuchiya, Sunarso, Riki Toita, Yoshihide Mori, Yasuharu Nakashima, Kunio Ishikawa

**Affiliations:** 1 Department of Biomaterials, Faculty of Dental Sciences, Kyushu University, 3-1-1 Maidashi, Higashi, Fukuoka 812-8582 Japan; 2 Section of Oral and Maxillofacial Surgery, Division of Maxillofacial Diagnostic and Surgical Sciences, Faculty of Dental Science, Kyushu University, 3-1-1 Maidashi, Higashi, Fukuoka 812-8582 Japan; 3 Department of Orthopaedic Surgery, Graduate School of Medical Sciences, Kyushu University, 3-1-1 Maidashi, Higashi, Fukuoka 812-8582 Japan; 4 Section of Bioengineering, Department of Dental Engineering, Fukuoka Dental College, 2-15-1 Tamura, Sawara, Fukuoka 814-0193 Japan; 5 Biomedical Research Institute, National Institute of Advanced Industrial Science and Technology (AIST), 1-8-31 Midorigaoka, Ikeda, Osaka 563-8577 Japan; 6 Present Address: Department of Oral Surgery, Institute of Biomedical Sciences, Tokushima University Graduate School, 3-18-15 Kuramotocho, Tokushima, 770-8504 Japan; 7 Present Address: Department of Dental Materials, Faculty of Dentistry, University of Indonesia, Jalan Salemba Raya No. 4, Jakarta, Pusat 10430 Indonesia

## Abstract

This study was aimed to investigate the osseointegration ability of poly(ether ether ketone) (PEEK) implants with modified surface roughness and/or surface chemistry. The roughened surface was prepared by a sandblast method, and the phosphate groups on the substrates were modified by a two-step chemical reaction. The *in vitro* osteogenic activity of rat mesenchymal stem cells (MSCs) on the developed substrates was assessed by measuring cell proliferation, alkaline phosphatase activity, osteocalcin expression, and bone-like nodule formation. Surface roughening alone did not improve MSC responses. However, phosphorylation of smooth substrates increased cell responses, which were further elevated in combination with surface roughening. Moreover, in a rabbit tibia implantation model, this combined surface modification significantly enhanced the bone-to-implant contact ratio and corresponding bone-to-implant bonding strength at 4 and 8 weeks post-implantation, whereas modification of surface roughness or surface chemistry alone did not. This study demonstrates that combination of surface roughness and chemical modification on PEEK significantly promotes cell responses and osseointegration ability in a synergistic manner both *in vitro* and *in vivo*. Therefore, this is a simple and promising technique for improving the poor osseointegration ability of PEEK-based orthopedic/dental implants.

## Introduction

Titanium and titanium alloy implants have been used in the fields of orthopedics and dentistry owing to their cytocompatibility, high mechanical strength, and excellent corrosion resistance^1^. However, their much higher elastic moduli compared with bone tissue as well as metal allergy cause implant failure from post-operative complications, such as metal allergy, osteolysis, and eventual loosening^2,3^. Another disadvantage of metallic implants is their potential artifacts or distortion in magnetic resonance imaging near the implantation site^4^. Poly(ether ether ketone) (PEEK) has distinct advantages over metallic implants, including radiolucency, excellent sterilization resistance, and cytocompatibility, and thus has received considerable attention as a potential substitute for metallic implants^5^. Moreover, the elastic modulus of PEEK (3–4 GPa) is more similar to that of cortical bone (18 GPa) than that of titanium alloy (110 GPa), and PEEK can be extensively tailored by preparing carbon-fiber reinforced composites^5^. Despite the many advantages of PEEK for application as an orthopedic and dental implant, its inert nature limits osseointegration and ultimately leads to implant subsidence and nonunion.

To overcome this clinical obstacle, PEEK/hydroxyapatite (HA) composites and HA-coated PEEK have been developed to take advantage of the highly osseointegration ability of HA^6–10^. These modified PEEK implants show excellent osseointegration ability; however, weak bonding between HA and PEEK has raised concerns about decreased mechanical properties and detachment of HA^5,6^. Thus, great efforts have been made to enhance osseointegration ability through modification of surface roughness and surface chemistry^11–24^. Surface roughness at the micron level plays a pivotal role in the osteogenesis of mesenchymal stem cells (MSCs) and osteoprogenitor cells *in vitro*^23–25^. Furthermore, bone tissue can infiltrate and grow into a roughened surface, thus enhancing the stability of implant fixation. However, these effects are material-dependent and have not been investigated well with respect to PEEK. Surface chemistry is another crucial factor for cell responses^13–16,18,19^. Recently, we and others have shown that phosphate group modification on titanium and its alloy significantly improves osteogenesis and osseointegration ability^15,16^.

Given that surface chemistry and roughness play distinct roles in osseointegration ability and implant fixation stability, we hypothesized that a combination of these surface modifications on PEEK would improve its osseointegration ability in a synergistic manner. Thus, a two-step method comprising phosphorylation with sandblasting was employed to modify the surface chemistry and roughness of PEEK, respectively. The effects of surface micro-roughening and phosphorylation on the osteogenic properties of rat MSCs, including proliferation, differentiation, and mineralization, were then examined. Additionally, we assessed various characteristics of osseointegration ability *in vivo*, including bone-implant contact and bonding strength, after implantation of the modified PEEK into rabbit tibia.

## Results

### Surface modification

**Figure 1:**
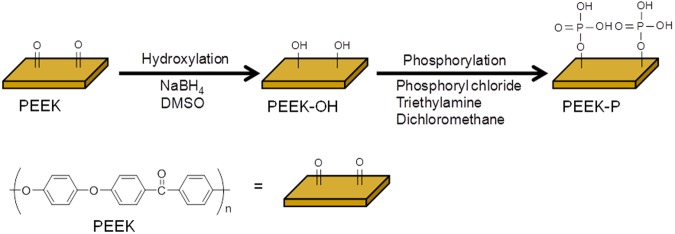
Schematic of PEEK sample preparation.

**Figure 2:**
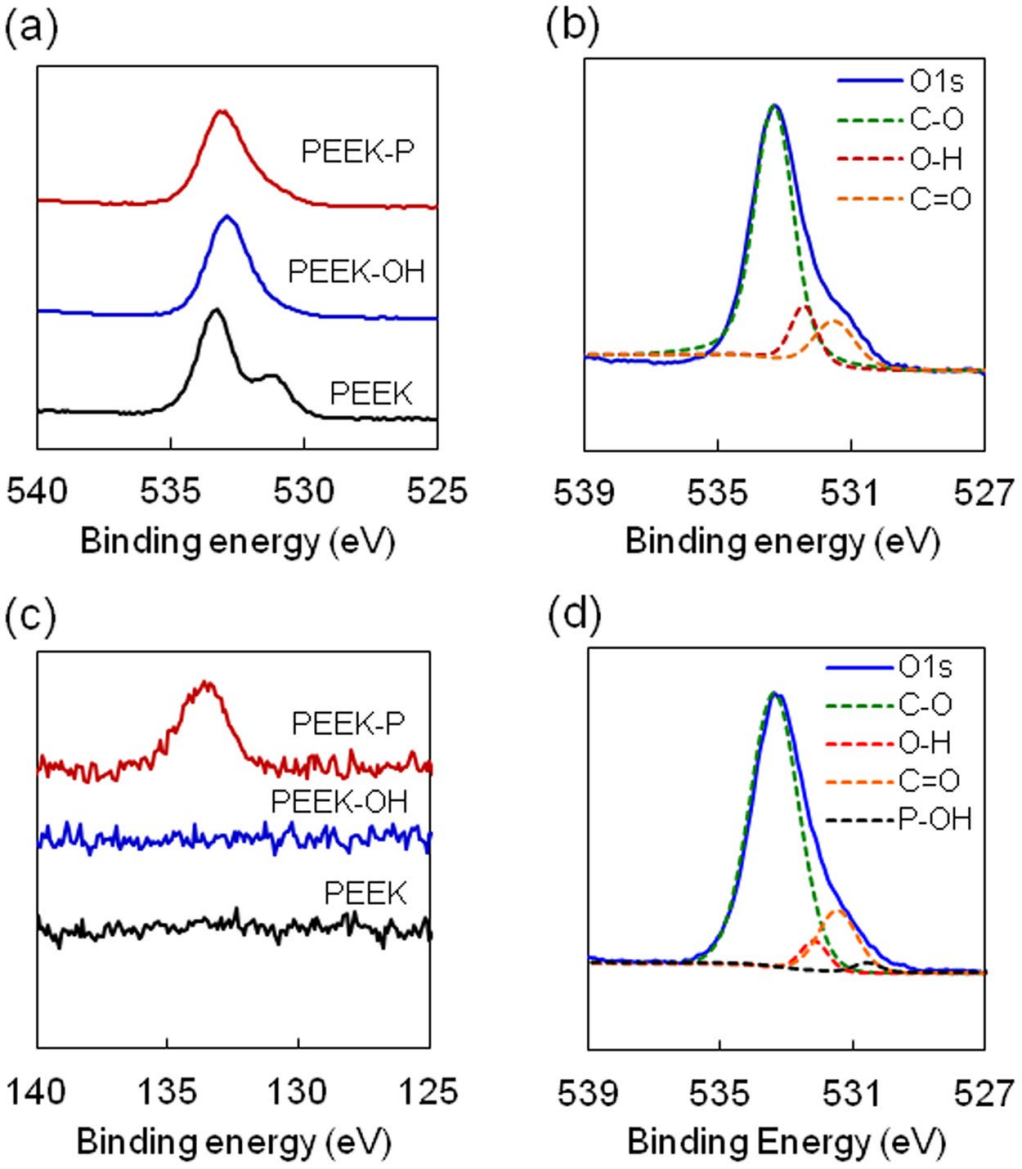
X-ray photoelectron spectroscopy (XPS) analysis. (**a**) O1s and (**c**) P2p spectra of PEEK samples. PEEK-OH and PEEK-P indicate hydroxylated and phosphorylated PEEK, respectively. (**b**) Peak separation of the O1s spectra of PEEK-OH. The hydroxyl group (-OH) was newly formed on PEEK-OH. (**d**) Peak separation of the O1s spectra of PEEK-P.

**Figure 3:**
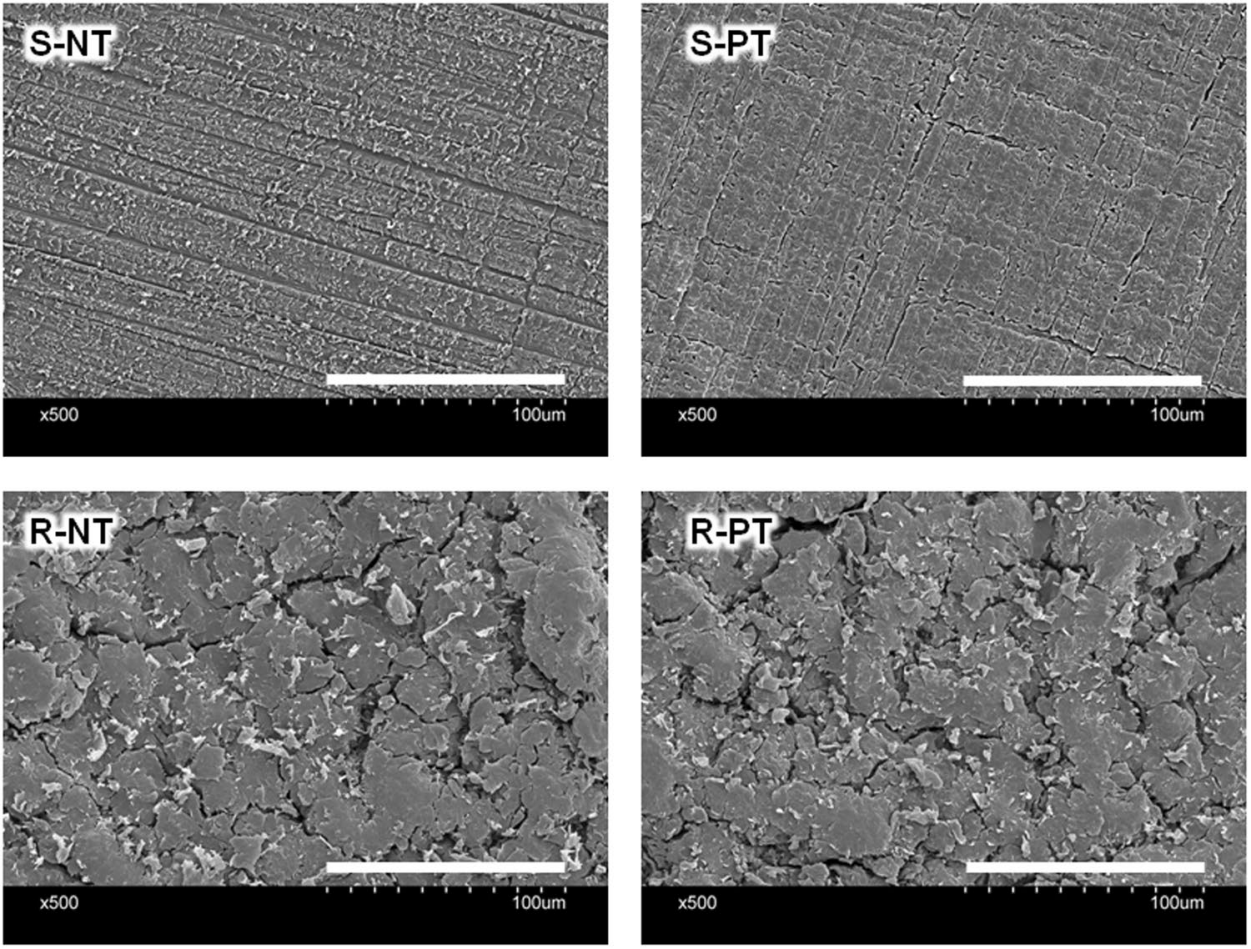
Scanning electron microscopy (SEM) observation of the PEEK sample surface. S-NT: untreated PEEK, S-PT: phosphorylated PEEK with smooth surface, R-NT: sandblasted PEEK, R-PT: phosphorylated PEEK with sandblasted surface.

We first investigated the effect of alumina size (F220-F60) on the mean roughness (*R*_a_) of sandblasted PEEK and found that F60 showed the largest *R*_a_ (Supplementary Fig. S1). Thus, in this study, alumina with a size of F60 was used as an abrasive to prepare the roughened PEEK. Phosphate group-modified PEEK was prepared by a two-step chemical reaction (Fig. 1). First, the carbonyl groups were reduced to hydroxyl groups using sodium borohydride to obtain PEEK-OH. Success of the preparation was confirmed by XPS analysis (Fig. 2a,b), whereby the carbonyl group (C=O), with a binding energy (BE) of 530.8–531.7 eV, was decreased in PEEK-OH, while a new peak, assigned as a hydroxyl group (-OH) was observed at a BE of 532.5 eV^26,27^. The C=O peak decreased and -OH peak increased as the reaction time increased, indicating that C=O was reduced to -OH (Supplementary Fig. S2). However, after 48 h of the reaction, a different surface topography was observed by scanning electron microscopy (SEM) compared to that of the original roughened PEEK (Fig. 3 and Supplementary Fig. S3) and *R*_a_ value was increased (2.6 ± 0.1 μm) compared to that of the original roughened PEEK (2.1 ± 0.1 μm). To compare the cell and tissue responses between bare and phosphorylated PEEK with similar surface topographies and *R*_a_ values, a reaction time of 24 h was used. After 24 h reaction, the ratio of hydroxyl groups in the total oxygen was estimated to be 11.4% (Table 1). PEEK-OH was further reacted with phosphoryl chloride, followed by hydrolysis of P-Cl bonds to obtain PEEK-P. XPS analysis revealed a newly generated P2p peak (BE = 134 eV) in PEEK-P (Fig. 2c). Additionally, the ratio of hydroxyl groups in the total oxygen was reduced from 11.4% to 7.9% after the phosphorylation reaction (Table 1 and Fig. 2d), and thus the conversion ratio was estimated to be approximately 30%. The atomic composition, water contact angle (CA), and *R*_a_ of the samples are summarized in Table 2. Phosphate group modification did not change the hydrophilicity of the surface regardless of surface roughness. Surface roughness after sandblast treatment, as observed by SEM (Fig. 3), showed that the *R*_a_ increased from <0.1 μm to 2.1 μm. The thicknesses of PEEK samples before and after sandblasting were 1,001 ± 5 and 996 ± 4 μm (n = 5), respectively, as determined with a micrometer. Phosphate modification barely changed the surface roughness and topography compared with the original samples.

**Table 1:** Relative composition of O1s in samples.

Binding energy (eV)	Attribution	Relative composition		
		PEEK	PEEK-OH	PEEK-P
530.6	P-OH	0	0	1.9 ± 0.5
531.3‒531.7	O=C	32.1 ± 1.0	13.1 ± 1.8	19.3 ± 6.3
532.4	O-H	0	11.4 ± 2.0	7.9 ± 3.7
533.3	O-C	67.9 ± 1.0	75.5 ± 2.2	70.8 ± 10

**Table 2:** Chemical and physical properties of PEEK samples.

^a^Samples	Atomic composition/At.%			^b^CA/°	^c^*R*_a_/μm
	C1s	O1s	P2p		
S-NT	87.5 ± 0.1	12.5 ± 0.1	—	104.1 ± 3.9	<0.1
S-PT	85.9 ± 1.0	13.7 ± 1.0	0.4 ± 0.1	96.6 ± 7.8	<0.1
R-NT	84.5 ± 0.9	15.5 ± 0.9	—	102.0 ± 5.9	2.1 ± 0.2
R-PT	83.1 ± 1.0	16.2 ± 0.9	0.7 ± 0.1	106.1 ± 13.9	2.1 ± 0.2

^a^S-NT: untreated PEEK, S-PT: phosphorylated PEEK with smooth surface, R-NT: sandblasted PEEK, R-PT: phosphorylated PEEK with sandblasted surface

^b^Static contact angle of water droplet.

^c^Mean roughness. The lower detection limit of the machine is 0.1 μm.

### Cell responses

**Figure 4:**
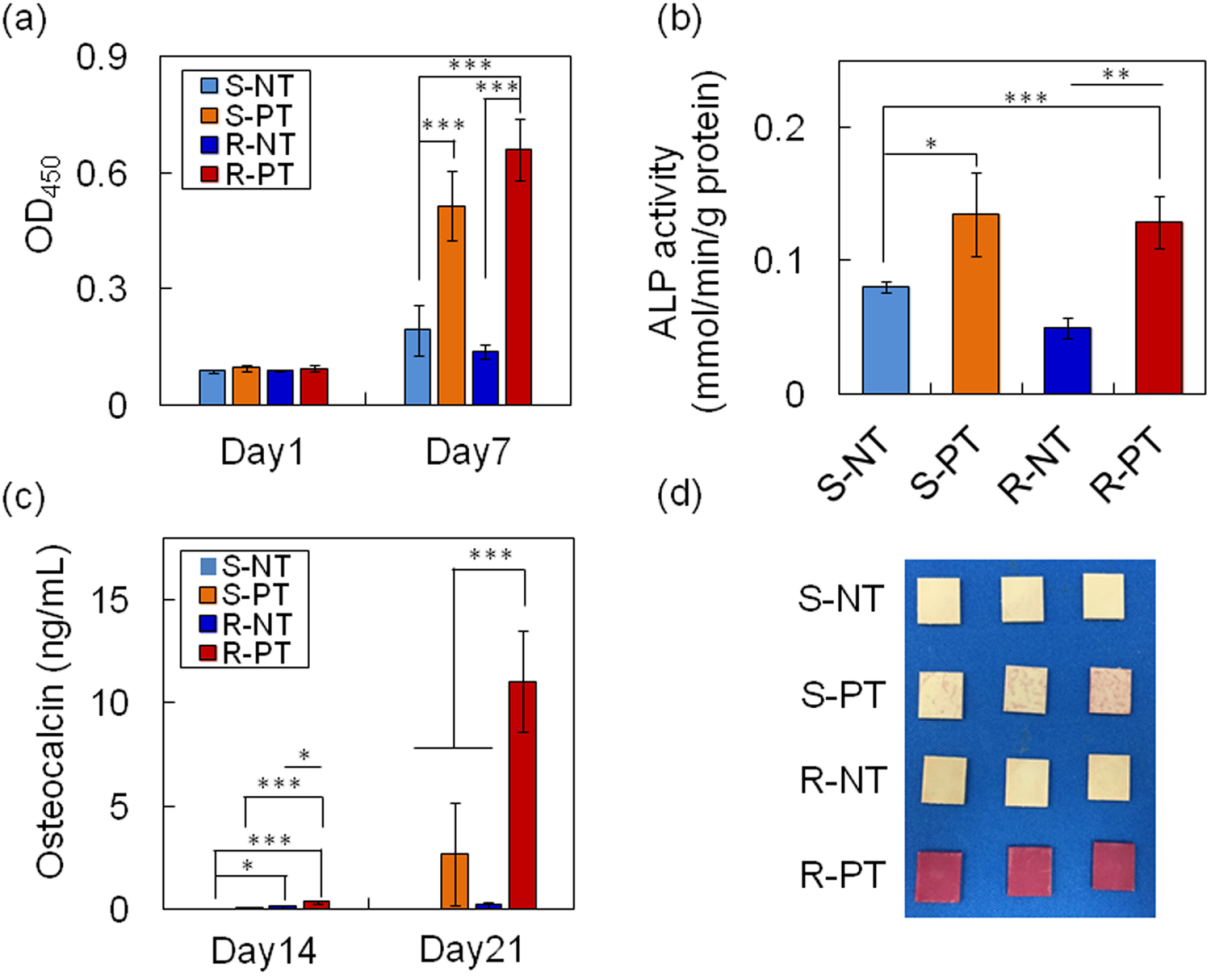
BMSC response on samples. (**a**) Cell proliferation on days 1 and 7. (**b**) Alkaline phosphatase (ALP) activity of the cells on samples at day 7. (**c**) Osteocalcin secretion from cells cultured on samples on days 14 and 21. Data shown are mean ± SD (*p < 0.05, **p < 0.01, ***p < 0.001). (**d**) Bone-like nodule formation on samples at day 28. Bone-like nodules were stained with alizarin red. S-NT: untreated PEEK, S-PT: phosphorylated PEEK with smooth surface, R-NT: sandblasted PEEK, R-PT: phosphorylated PEEK with sandblasted surface.

Rat BMSCs, undergo multilineage differentiation into a variety of cells, including osteoblasts, therefore were used to compare the osteoconductive properties of the samples. On day 1, the numbers of viable cells on the samples were similar (Fig. 4a). On day 7, the phosphate-modified smooth and rough PEEK samples (S-PT and R-PT, respectively) showed increased cell proliferation compared with the unmodified smooth and rough samples (S-NT and R-NT, respectively); however, surface roughness did not affect cell proliferation. A similar trend was observed for ALP activity, a marker of early stage differentiation (Fig. 4b). Osteocalcin levels increase at the later stages of differentiation. Low levels of osteocalcin were observed in all samples on day 14, although R-PT exhibited the highest osteocalcin levels (Fig. 4c). On day 21, much higher levels of osteocalcin were observed in the phosphate-modified samples (S-PT and R-PT) than the unmodified samples (S-NT and R-NT). Surface roughening of the phosphate-modified samples further activated osteocalcin secretion, as observed in R-PT. Mature osteoblasts form bone-like nodules, however on day 28, only R-PT exhibited bone-like nodule formation, whereas in the other samples, nodule formation was negligible (Fig. 4d).

### Tissue responses

**Figure 5:**
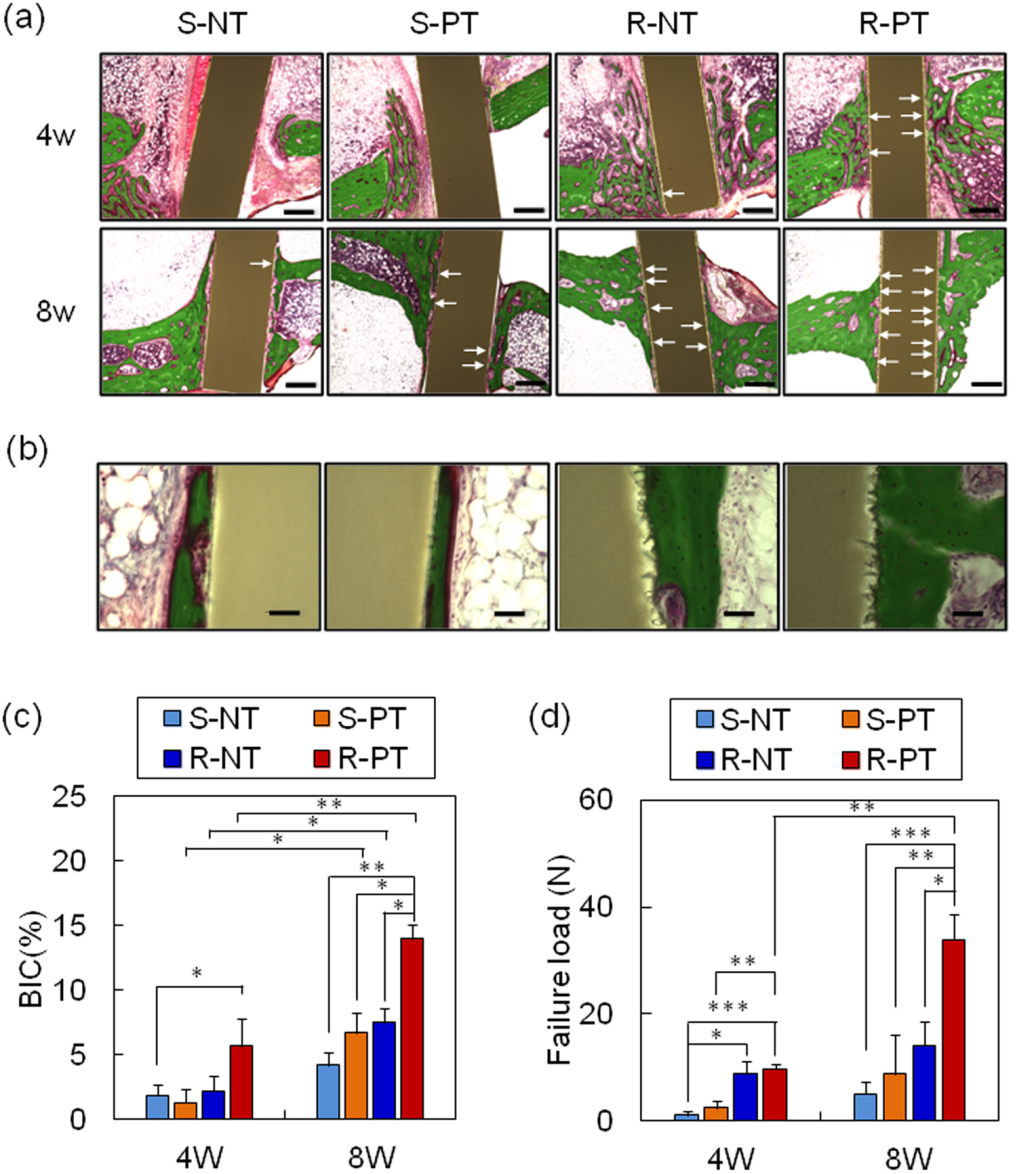
Rabbit tibia implantation. (**a**,**b**) Representative histological images of new bone formation and osseointegration of samples at 4 and 8 weeks post-implantation. White arrows indicate new bone tissue in direct contact with the PEEK samples. Scale bars are (**a**) 500 μm and (**b**) 50 μm. S-NT: untreated PEEK, S-PT: phosphorylated PEEK with smooth surface, R-NT: sandblasted PEEK, R-PT: phosphorylated PEEK with sandblasted surface. (**c**) Bone-to-implant contact (BIC) ratio (n = 4) and (**d**) failure load between bone and PEEK samples (n = 6) at 4 and 8 weeks post-implantation. Data are mean ± SE (*p < 0.05, **p < 0.01, ***p < 0.001).

Osseointegration, defined as the direct contact between the implant and bone, without fibrous tissue growth between the interface, is crucial for the stability of implant-to-bone fixation^28^. To compare the osseointegration ability of modified and unmodified PEEK, specimens were implanted into rabbit tibia, and the bone-to-implant contact ratio (BIC; Fig. 5a‒c) and bone-implant bonding strength (failure load between the bone and implant; Fig. 5d) were determined at 4 and 8 weeks post-implantation. At 4 weeks, new bone was formed surrounding the old bone in all samples. The BIC of S-PT and R-NT was similar to that of S-NT. The BIC of R-PT was two folds higher than the BIC of the other samples. In accordance with this result, the failure load of R-PT was higher than those of S-NT and S-PT. At 8 weeks, new bone growth enhanced in the area surrounding the samples. The BIC of the treated samples at 8 weeks was higher than the BIC at 4 weeks; however, the BIC of S-NT did not increase between 4 and 8 weeks post-implantation (*p* > 0.05), indicating poor osseointegration ability. R-PT exhibited the greatest BIC among the treated samples. Moreover, R-PT showed greatly enhanced failure load compared with S-NT, whereas failure loads of the other samples were similar (*p* > 0.05). These results demonstrate that modified surface chemistry and roughness synergistically affect the osseointegration ability and bone fixation of an implant, with single-surface modification being insufficient to improve such properties.

## Discussion

Surface roughness and chemical modification of biomaterials affect cell behaviors; thus, considerable attention has been paid to the application of such techniques where tissue fixation is critical^11–24^. Despite numerous publications demonstrating the effect of single-modification on cell and tissue responses, including surface roughness or chemical based, limited studies exist on the effect of combined modifications, especially on tissue responses. Therefore, we prepared a series of PEEK implants with distinct surface roughness and surface chemistry modifications, and compared the osteogenesis of MSCs and osseointegration ability in the rabbit tibia using these implants. The major finding of this study was that the combined surface modifications in surface chemistry and topography of the PEEK implants are essential to elevate MSC osteogenesis *in vitro* and osseointegration ability in a rabbit tibia implantation model.

With advances in surface engineering research, well-defined micro- or nano-topographical surface modifications (e.g., grooves and pits) can be generated by physical and chemical roughening, pattern-transfer, and lithography^17^. *In vitro* studies have demonstrated that MSCs and osteoprogenitor cells exhibit different responses depending on surface topography via integrin signaling, focal adhesion, actin reorganization, and downstream signal/gene activation for osteogenesis, including c-Src, rho-associated protein kinase, focal adhesion kinase, ERK1/2, and Runx-2^17^. Until now, however, the effect of this on *in vivo* osseointegration ability has not been extensively studied. In contrast, sandblast technology is simple, cost-effective, and is already used on clinical metal implants^29^; however, *in vivo* osseointegration ability of the sandblasted PEEK has not been extensively studied^24^, which prompted us to apply this technology to PEEK in this study. However, we observed that sandblast treatment alone (R-NT) did not increase osteogenesis in rat-derived MSCs although a slight increase in the osteocalcin level was observed on day 14 (Fig. 4). In our previous study, however, R-NT enhanced cell proliferation, osteocalcin level, and bone-like nodule formation compared to S-NT^24^. These differences may be related to the following differences in the assay protocols: (1) number of media exchanges, (2) fetal bovine serum (FBS) concentration in the medium, and (3) initial cell density seeded on the samples. In this study, the number of media exchanges, FBS concentration, and initial cell density were decreased from three times/week to two times/week, from 15% to 10%, and from 4.0 × 10^4^ to 1.0‒2.5 × 10^4^, respectively. Cells require nutrients such as growth factors and amino acids to grow and differentiate. The initial cell density seeded on the samples affects cell growth and differentiation because cells grow exponentially depending on the cell density, and cell-cell contact is essential for inducing differentiation to mature osteoblasts, as discussed in detail below. Particularly, decreasing these factors significantly attenuated bone-like nodule formation by MSCs as observed for S-NT and R-NT. A study of MSCs on polycaprolactone with a surface roughness of 0.5–4.7 μm, showed that a surface roughness of 2.1‒3.1 μm was optimal for osteogenesis^30^. On titanium alloys with a surface roughness of 0.32‒0.87 μm, MSC ALP activity increased with increasing roughness^31^, whereas no increase in ALP activity was observed on HA with surface roughness in the range of 0.73‒4.68 μm^32^. These previous results indicate that improved osteogenesis, imparted by modulating surface roughness is material-dependent. Thus, systematic *in vitro* studies are required to determine whether surface roughness improves the osteoconductivity of PEEK and find optimal surface roughness.

Various methods, such as poly (dopamine) (pD) and poly (norepinephrine) (pNE) coating^18^, plasma treatment^33^, and chemical treatment using diazonium chemistry^14^, sodium borohydride reduction^26,27^, and photo-initiated polymerization^22^, have been used to successfully alter the surface chemistry of PEEK. pD and pNE can be coated on various materials, such as metals, ceramics, and polymers via non-covalent bonding^34^. These coatings can be used to modify chemicals with phosphate and (bis) phosphonate functional groups via Michael addition reaction. In our previous reports, PEEK coated with pNE enhanced the osteogenesis of MC3T3-E1 mouse pre-osteoblasts and human MSCs^18^. However, the pNE coating showed only a limited increase in bone-implant bonding strength, because the weak bonding strength between the PEEK surface and pNE caused detachment of the pNE coating from the implants (data not shown); indicating that alternative modification methods that not rely on coating is required.

Given the evidence that phosphate surface-modification enhanced the osteoconductivity of titanium implants in our previous study^15^, we used this strategy for PEEK implants, on which the phosphate groups were modified via a covalent bond. PEEK has ketone groups that can be reduced to hydroxyl groups by sodium borohydride^26,27^, and the resulting hydroxyl groups can be further reacted with phosphoryl chloride to prepare phosphate group-modified PEEK. The conversion ratio of hydroxyl groups to phosphate groups was approximately 30% according to XPS analysis. Similarly, Noiset *et al*. reported that 30% and 48% of hydroxyl groups on hydroxylated PEEK reacted with *p*-nitrophenyl chloroformate and acetic anhydride, respectively^27^. Acetic anhydride, which is smaller than phosphoryl chloride and *p*-nitrophenyl chloroformate, showed a higher conversion ratio. Thus, because of the relatively large size of phosphate, its presentation on PEEK may sterically hinder the reaction of phosphoryl chloride with hydroxyl groups on hydroxylated PEEK, resulting in a moderate conversion ratio of hydroxyl groups to phosphate groups.

Similar to phosphate-modified titanium implants, surface phosphorylation of PEEK enhanced MSC proliferation and ALP activity as compared to corresponding non-phosphorylated PEEK (Fig. 4). Interestingly, modifications of phosphate-groups and surface topography (R-PT) elevated osteocalcin levels and bone-like nodule formation by MSCs in a synergistic manner. On cell-cell contact, cells can exchange intracellular bioactive molecules, such as ions and second messengers, via gap junctions to facilitate differentiation. Inhibition or disruption of connexin 43, one of the components of gap junctions, decreases osteogenic differentiation and bone-like nodule formation^35^. In addition, MSCs have been shown to increase ALP activity as cell number increases (1–9 cells/microdomain) because cell-cell contact can be achieved at earlier time points if the MSCs are cultured at high density^36^. Thus, the increased osteogenesis observed with phosphorylated PEEK is likely due to increased cell proliferation. Another possibility is related to the phosphate groups on phosphorylated PEEK, as phosphate ion is essential for elevating differentiation markers, such as runt-related transcriptional factor 2 (Runx-2), osteopontin levels, and bone-like nodule formation by osteoprogenitor cells^37–39^. Such osteogenic markers can be activated by the recognition of increased extracellular concentrations of phosphate ions by the phosphate transporter expressed on the cell surface^40,41^. Nano-sized HA (nHA) was shown to enhance MSC osteogenesis with some of upregulated and downregulated genes overlapping with those observed following stimulation with 4–10 mM of phosphate ions^42–44^. However, elevated levels of osteogenic markers induced by nHA were dramatically decreased when the phosphate transporter was blocked with an inhibitor^42^. This may be because nHA dissolution increased the extracellular concentration of phosphate ions surrounding MSCs. Similar to nHA, phosphate groups on phosphorylated PEEK may be partially hydrolyzed, increasing the concentration of phosphate ions. Hence, BMSCs attached to phosphorylated PEEK may be exposed to an increased concentration of phosphate ions, resulting in enhanced osteogenesis.

Finally, we compared osseointegration ability of the PEEK implants with respect to BIC and bonding strength between bone tissue and implants using rabbit tibia implantation model (Fig. 5). Phosphorylation alone (S-PT) failed to improve both BIC and bonding strength although *in vitro* results revealed that proliferation and ALP activity of MSCs on S-PT were larger than those on bare PEEK (S-NT). Similarly, in agreement with the *in vitro* results, surface topography alone (R-NT) showed negligible improvement except for bonding strength at 4 weeks post-implantation. In a rat bone marrow implantation model, a higher bonding strength was also observed from R-NT than from S-NT at 4 weeks post-implantation^24^. As reported previously^21,25^, bone tissue could infiltrate and grow into the roughened surface prepared on R-NT, thereby enhancing stability of implant fixation. However, at 8 weeks after implantation, there was no significant difference in bonding strength between R-NT and S-NT. In contrast to R-NT and S-PT, R-PT maintained elevated BIC and bonding strength over an 8 week period. Given that MSCs showed the greatest osteogenesis on R-PT *in vitro* (Fig. 4), R-PT might provide a favorable environment for endogenous MSCs to grow and differentiate, thus permitting new bone formation inside the roughened surface over longer time periods. Thus, we suggest that combined modification of both surface topography and surface chemistry on PEEK implants was found to be essential to increase BIC and bonding strength *in vivo*, where surface topography and surface chemistry can provide enhanced effects on bonding strength and osteogenic activity of MSCs, respectively. However, further investigation is required to determine optimal surface topography, density of phosphate groups and the mechanism underlying the improved osseointegration ability of R-PT observed *in vivo*.

## Conclusions

We successfully prepared series of PEEK implant with distinct surface topography and surface chemistry. Modification of surface topography alone failed to improve MSC osteogenesis although a slight increase in the osteocalcin level was observed on day 14. Although modification of the surface topography alone increased the *in vivo* bone-implant bonding strength at 4 weeks after implantation compared to non-modified PEEK, strength did not significantly differ at 8 weeks. Modification of the phosphate-groups alone did not improve osseointegration ability, although this modification improved MSC responses, including proliferation and ALP activity *in vitro*. Importantly, the combination of these modifications resulted in significant elevation of MSC activity, especially with regard to osteocalcin expression and bone-like nodule formation, as well as osseointegration ability *in vivo*. This simple and non-coating modification is a promising technique for improving the poor osseointegration ability of PEEK; although further studies are needed to elucidate the mechanisms underlying the improved osseointegration ability of PEEK via phosphate groups and micro-topography.

## Materials and Methods

### Sample preparation

Square PEEK plates (10 × 10 × 1 mm; Ensinger Japan Co., Ltd., Tokyo, Japan) were used in this study. The PEEK substrates were polished with SiC paper (#800) and ultrasonically washed with ultrapure water, ethanol, and acetone for 30 min. Then, the surface was roughened by sandblasting using alumina particles (F60–F220) with a pressure of 0.5 MPa in for 10 s, and ultrasonically washed with acetone and water. The PEEK samples were reacted with sodium borohydride (57 mg, 1.5 mmol; Tokyo Chemical Industry Co., Ltd., Tokyo, Japan) dissolved in 30 mL of dimethylsulfoxide (Wako, Osaka, Japan) at 80 °C for 24 h, and then soaked in 0.5 M HCl (Wako) and water to produce PEEK-OH. PEEK-OH was soaked in 20 mL of dichloromethane (Wako) containing phosphoryl chloride (307 mg, 2 mmol; Wako) and triethylamine (202 mg, 2 mmol; Wako) with shaking (90 rpm) for 24 h at room temperature. After washing the treated substrates with acetone, they were hydrolyzed with ultrapure water for 30 min at room temperature to prepare phosphate-modified PEEK. Finally, the substrates were washed with ethanol and acetone, and then dried under nitrogen.

### Surface characterization

Surface topography was observed by SEM (S-3400N; Hitachi High Technologies Co., Tokyo, Japan) under an accelerating voltage of 5 kV after coating with gold. Surface chemical composition was evaluated by X-ray photoelectron spectroscopy (XPS, K-alpha; Thermo Fisher Scientific, East Grinstead, UK). Au 4f_7/2_ with a BE of 83.96 eV and Ag 3d_5/2_ with a BE of 368.21 eV were used to calibrate the XPS system. In addition, the maximum peaks of C1s for each measurement were set to 284.8 eV. The CA of each sample droplet (1.5 μL) was measured using a CA meter (DM500; Kyowa Interface Science, Saitama, Japan). Surface roughness was measured by laser scanning microscopy (Violet Laser VK-9700; Keyence, Osaka, Japan).

### Cell culture

Rat bone marrow-derived stem cells (rBMSCs) were obtained from 8-week-old, male Wistar rats (Japan SLC, Shizuoka, Japan) and were cultured in minimum essential Eagle’s medium, α-modification (MEMα; Wako) supplemented with 10% fetal bovine serum (FBS), 100 U/mL penicillin, 100 mg/mL streptomycin, and 0.25 mg/mL amphotericin B (all from Gibco, Grand Island, NY, USA). The cells were cultured in cell flasks at 37 °C in a humidified atmosphere of 5% CO_2_ and 95% air. The medium was replaced every two days. On reaching 80% confluence, the cells were used experimentally.

### Cell proliferation

PEEK samples were sterilized with 70% ethanol and dried under N_2_ gas before experimentation. Samples were placed in a 24-well cell culture plate, and the cells were seeded on the samples at an initial density of 1.0 × 10^4^ cells/well. On days 1 and 7, cell numbers were estimated using the Cell Counting Kit 8 (CCK-8; Dojindo, Kumamoto, Japan) according to the manufacturer’s protocol. Absorbance at 450 nm was measured using a microplate reader (Infinite M200; TECAN, Victoria, Australia).

### Determining alkaline phosphatase (ALP) activity, osteocalcin expression, and bone-like nodule formation

Samples were placed in 24-well cell culture plates, and cells were seeded on the samples at cell density of 2.5 × 10^4^ cells/well. The initial medium was exchanged for differentiation medium (culture medium containing 10 mM β-glycerophosphate (Wako) and 10 nM dexamethasone (Wako)) twice a week. On day 7, the cells were washed three times with PBS and lysed in 500 μL of lysis buffer M (Wako; containing 20 mM Tris-HCl, 200 mM sodium chloride, 2.5 mM magnesium chloride, and 0.05% NP-40 substitute). ALP activity was measured using the LabAssay ALP Kit (Wako). Absorbance at 405 nm was measured with a microplate reader. ALP activity was standardized according to the protein concentration in the lysate, which was measured by the Bradford method using the Bio-Rad Protein Assay Kit (Bio-Rad Laboratories, Inc., Hercules, CA, USA).

On days 13 and 20, the medium was refreshed and then collected 24 h later. Collected medium was frozen at −80 °C until measurement. Osteocalcin concentration was measured using the Rat Gla-Osteocalcin High Sensitive EIA kit (Takara BIO, Inc., Shiga, Japan).

On day 28, bone-like nodules were visualized using the Bone Nodule Staining kit (Cosmo Bio Co., Ltd., Tokyo, Japan) containing Alizarin red S. Samples were washed three times with PBS. The cells were then fixed with −20 °C methanol at room temperature for 30 min and then washed with water. Chromogenic substrate solution (400 µL) was dispensed in each well and incubated for 5 min, and then washed with substrate-containing buffer until the washing solution was colorless.

### Animal experiment

Male Japanese white rabbits (aged 19–20 weeks; mean body weight, 2953 ± 129 g) were purchased from Japan SLC (Shizuoka, Japan). Rabbits were single housed in standard cages and were maintained in a temperature-controlled room (22 °C) under a 12-h light-dark cycle. Rabbits were fed a normal diet (CRF-1; Oriental Yeast Co., Ltd., Tokyo, Japan) and sterilized tap water *ad libitum* over the course of the experiment. A total of 40 rabbits were used and 80 samples were implanted in the animals. In each rabbit, two PEEK plates (10 × 10 × 1 mm) were implanted in the tibial bone as described below. The animal study protocols were approved by the Ethical Committee for Animal Experimentation at Kyushu University (Admission number: A28-061-0), and the experiments were performed in accordance with the Guidelines for the care and use of Laboratory animals established by established by the National Institute of Health.

### Sample implantation

The rabbits were anesthetized by intraperitoneal injection of ketamine (30 mg/kg) and xylazine (5 mg/kg). The surgical area was shaved and cleaned with iodine before surgery. After administration of 1 wt% lidocaine as a local anesthetic, a 3-cm longitudinal skin incision was made on the medial side of the knee, followed by incision of the fascia and periosteum to expose the tibial cortex. An approximately 10 × 1.3 mm slit-like perforation was cut using a dental bur in the proximal metaphysis of the tibia in parallel to the longitudinal axis of the tibia. After the hole was irrigated with sterile saline, the PEEK plates (10 × 10 × 1 mm) were implanted. The fascia and skin were repositioned and sutured layer by layer. After 4 and 8 weeks, the rabbits were euthanized by administration of an overdose of ketamine/xylazine, and the bone tissues were harvested and analyzed in the following experiments.

### Biomechanical testing

To determine the failure load between the bone tissue and PEEK plate, a detachment test was performed as described previously^45^, with slight modification. The tissue was adjusted until it was vertically aligned with the load cell. The failure load was determined using a universal testing machine (Autograph AGS-J; Shimadzu, Japan), with a calibrated load-cell of 1 kN. The cross-head speed was set to 10 mm/min. The failure load was defined as the load at which the sample detached from the bone tissue.

### Histological analysis

The bone tissue was fixed with 70% ethanol and dehydrated in a graded series of ethanol. The tissue was then embedded in poly (methylmethacrylate) by polymerization of methylmethacrylate (Nacalai Tesque, Inc., Tokyo, Japan) and dimethyl 2,2’-azobisisobutyrate (Wako) as an initiator at 37 °C for 7 days. Thick tissue sections (1 mm) were cut with a diamond cutter and ground to a final thickness of ~60 μm using a grinding unit. The sections were subjected to Villanueva-Goldner staining, and histological examination was performed using All-in-One Fluorescence Microscopy (BZX710; Keyence). Bone implant contact (BIC) was calculated as: (length of mineralized bone in direct contact with PEEK/total PEEK length) × 100.

### Statistical analysis

The mean and standard deviation (SD) or standard error of mean (SE) of the data were calculated. The statistical significance between test groups was evaluated using one-way analysis of variance (ANOVA) and Tukey’s multiple comparison test. A *p* value less than 0.05 was considered significant.

## Electronic supplementary material

Supplementary information

## Data Availability

The datasets generated during and/or analysed during the current study are available from the corresponding author on reasonable request.
