# Supplementary material for: Synergistic effect of surface phosphorylation and micro-roughness on enhanced osseointegration ability of poly(ether ether ketone) in the rabbit tibia
Source: Sci Rep. 2018 Nov 15;8:16887. doi: 10.1038/s41598-018-35313-7 (PMC6237893; doi:10.1038/s41598-018-35313-7)
Supplement: Supplementary file 1 — Supplementary information [file 41598_2018_35313_MOESM1_ESM.pdf]

Supplementary information

Synergistic effect of surface phosphorylation and micro-roughness on enhanced osseointegration ability of poly(ether ether ketone) in the rabbit tibia

Naoyuki Fukuda, Masayuki Kanazawa, Kanji Tsuru, Akira Tsuchiya, Sunarso, Riki Toita, Yoshihide Mori, Yasuharu Nakashima & Kunio Ishikawa

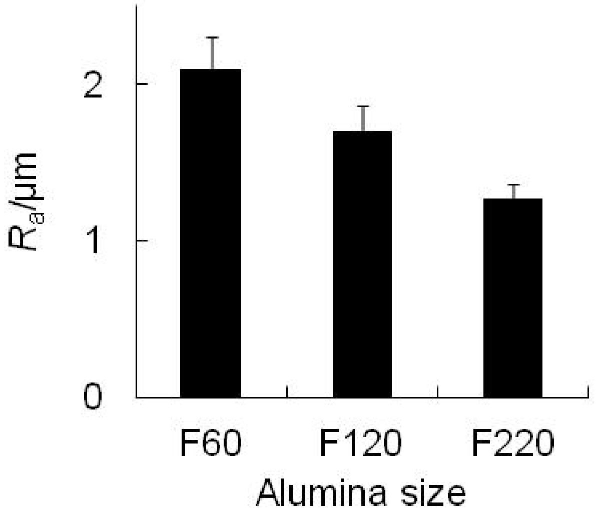

Supplementary Fig. S1. R_a values of PEEK sandblasted by different alumina size.

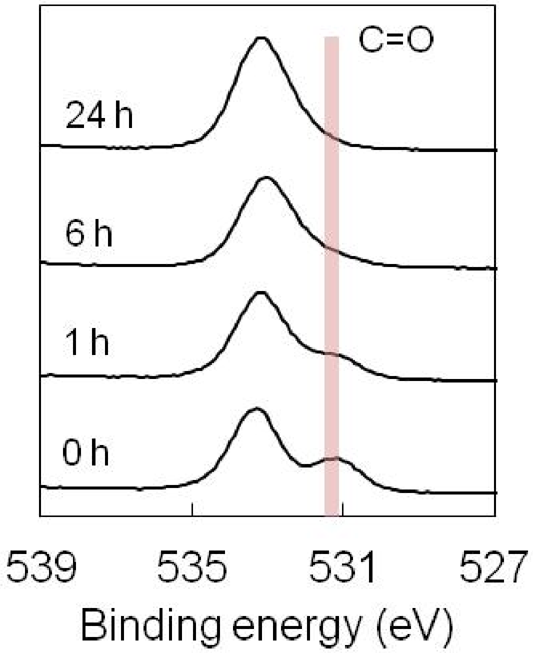

Supplementary Fig. S2. O1s spectra of hydroxylated PEEK by $NaBH_4$ reduction reaction. Intensity of C=O peak decreased as increase in reaction time.

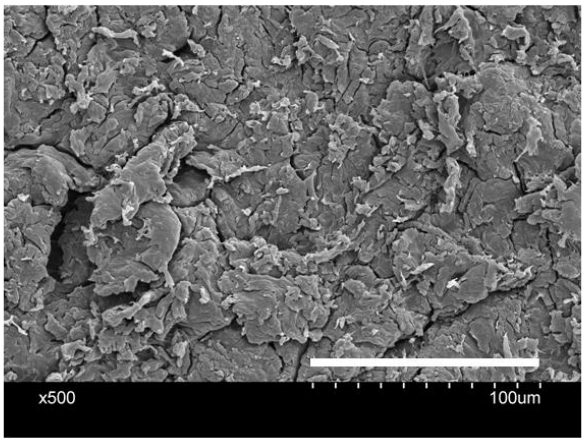

Supplementary Fig. S3. SEM image of roughened PEEK at 48 h after $NaBH_4$ reaction.
